# Molecular dissection of ependymomas

**DOI:** 10.18632/oncoscience.202

**Published:** 2015-08-20

**Authors:** Kristian W. Pajtler, Stefan M. Pfister, Marcel Kool

**Affiliations:** Division of Pediatric Neurooncology, German Cancer Research Center DKFZ, Heidelberg, Germany

**Keywords:** ependymoma, brain tumor, molecular classification

Ependymal tumors comprise a heterogeneous group of neuroepithelial malignancies of the CNS with variable prognosis that can occur in children and adults along the entire neuroaxis, including the spine (SP), posterior fossa (PF), and supratentorial brain regions (ST). Currently, these tumors are classified and graded solely by morphological patterns, but it is commonly accepted that grading does not accurately predict their clinical behavior [1]. A powerful clinical stratification system is thus lacking to date. In a recent paper, we aimed to address this challenge by developing an unbiased, robust and uniform molecular classification of ependymal tumors that adequately reflects the full biological, clinical and histopathological heterogeneity across all age groups, grades and major anatomical CNS compartments [2].

DNA methylation patterns in tumors have been shown to represent a very stable molecular memory of the respective cell of origin throughout disease course, making them particularly suitable for tumor classification purposes. We generated genome-wide DNA methylation profiles for 500 ependymal tumors using the Illumina 450k methylation array and identified nine distinct molecular subgroups, three within each CNS compartment. One of the subgroups within each compartment was enriched with grade I subependymomas (SE), named SP-SE, PF-SE and ST-SE. Other molecular subgroups within the spine showed a relatively good concordance with the histopathological subtypes myxopapillary ependymoma (SP-MPE) and (classic) ependymoma (SP-EPN). The remaining molecular subgroups within the posterior fossa were the previously described PFA and PFB ependymomas [3,4], renamed for consistency as PF-EPN-A and PF-EPN-B. One supratentorial subgroup, termed ST-EPN-RELA, was characterized by quasi-defining *C11orf95- RELA* gene fusions, recently identified by Parker et al. [5] to occur in around 70% of ST ependymomas. Our molecular classification now shows that this is a distinct molecular subgroup with poor outcome in which almost all tumors harbor this fusion. *RELA* gene fusions were not found in any of the other subgroups. In a second ST subgroup (ST-EPN-YAP1) with good outcome we identified highly recurrent *YAP1* fusions as the probably most important driver event, again not present in any of the other subgroups. We found no other recurrent gene fusions in the other subgroups, confirming previous sequencing studies [5,6]. These sequencing studies also revealed that ependymomas have overall very few mutations and in PF ependymomas no recurrent genetic hits were found thus far, suggesting that either epigenetic, copy number, or other structural alterations may drive these tumors.

**Figure 1:**
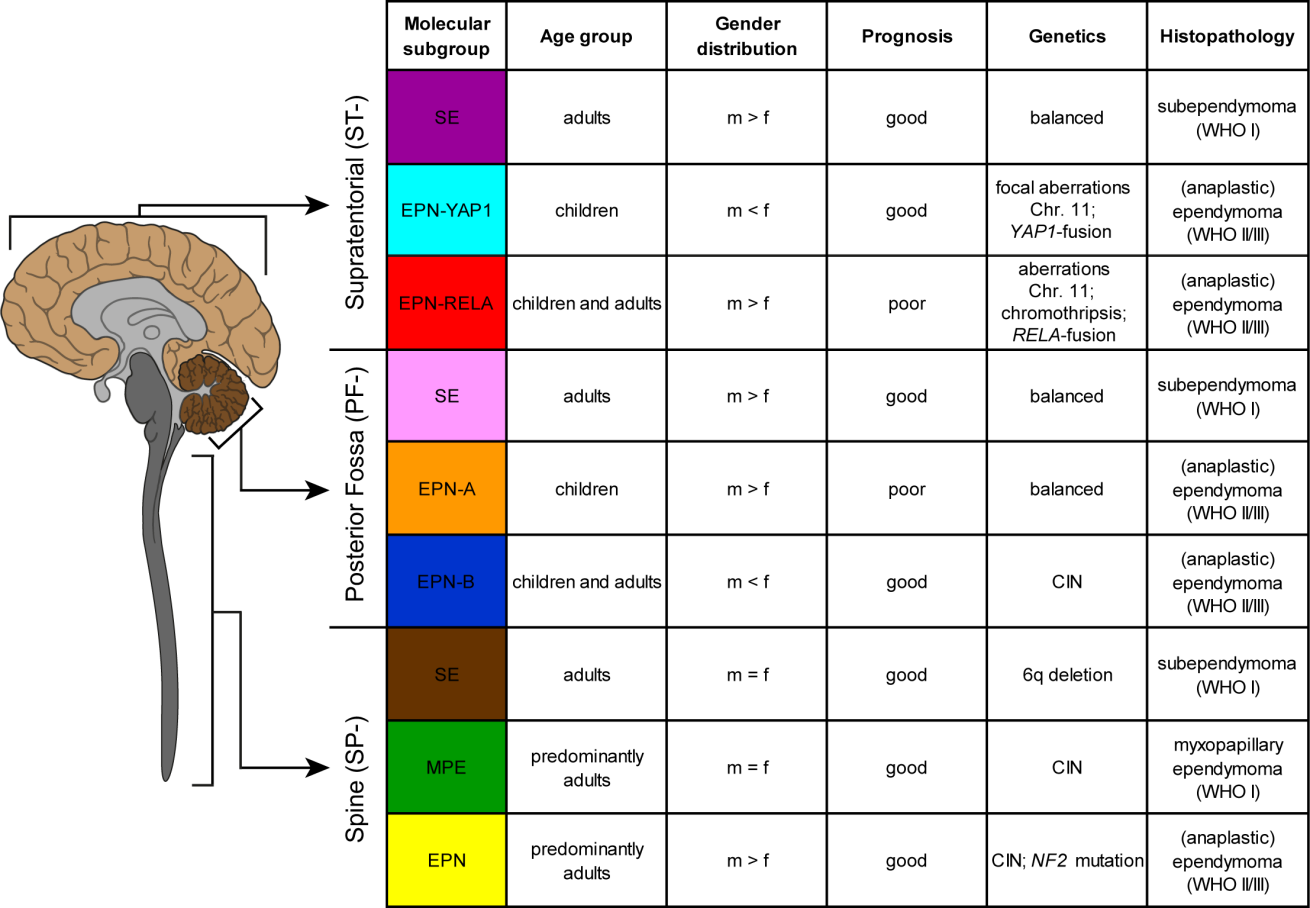
Table shows key genetic, epigenetic, demographic and clinical findings in the nine molecular subgroups of ependymal tumors as identified by methylation profiling CIN, Chromosomal instability.

The molecular subgroups identified in our study were closely associated with specific age groups. Tumors of the PF-EPN-A subgroup were almost exclusively found in young children and also ST subgroups, ST-EPN-YAP1 and ST-EPN-RELA, were much more common in children. Tumors in the other subgroups were more common or exclusively found in adults. Patients with PF-EPN-A and ST-EPN-RELA tumors, together comprising two-thirds of all cases, have the worst outcome. In contrast, patients in the other subgroups have an excellent prognosis with 5-year overall survival rates close to 100%. Multivariable analysis identified molecular subgroup, level of resection and gain of chromosome arm 1q to be of independent prognostic value for both overall and progression free survival but did not show any predictive impact of WHO-grading [2].

Genome-wide DNA copy number profiles showed strong differences of DNA copy number alterations (CNAs) between the nine molecular subgroups. Most CNAs across all subgroups involved gains or losses of whole chromosomes compatible with aneuploidy. In accordance with findings by Parker et al. [5], we frequently observed patterns of chromothripsis in ST-EPN-RELA tumors, mainly involving chromosome 11, but never in any other subgroup. In contrast, ST-EPN-YAP1 tumors frequently displayed focal CNAs around the *YAP1* locus, which is also on chromosome 11, while the remainder of the genome appeared to be relatively stable. Predominantly stable genomes were also found for all three molecular subependymoma subgroups (SP-SE, PF-SE, ST-SE) as well as for PF-EPN-A. The most frequent event in the latter subgroup was gain of chromosome 1q, which is an established marker for poor outcome in ependymoma [7]. Spinal subgroups SP-MPE and SP-EPN displayed various CNAs, frequently involving the 22q locus in SP-EPN tumors which includes a known oncogenic driver of this subgroup, *NF2*. The PF-EPN-B subgroup showed by far the highest degree of genomic instability, with many gains and losses of entire chromosomes or chromosomal arms in each tumor. Taking into account that CNAs are not occurring randomly and therefore presumably contain drivers of cancer, Mohankumar and colleagues recently reported a cross-species *in vivo* screen of 84 candidate oncogenes and 39 candidate tumor suppressor genes (TSG), located within 28 recurrent CNAs in ependymoma [8]. The authors identified eight new ependymoma oncogenes and ten new ependymoma TSGs, which converged on dysregulation of specific cell functions, including trafficking of growth factor receptors, FGFR and EGFR, known to be oncogenic in ependymoma. It will now be interesting to see whether any of these newly identified ependymoma oncogenes or TSGs may generate ependymomas faithfully modeling distinct subgroups.

In summary, we have described a new, comprehensive and robust classification of ependymal tumors based on DNA methylation profiling. Importantly, we have also shown that subgroup classification remains stable at the time of recurrence [2]. Future application of the classification in a clinical setting might enable to assess treatment efficacies in the context of specific molecular subgroups thereby refining current treatment approaches or allowing for implementation of targeted therapies. Since the proposed risk stratification based on molecular subgrouping is superior to histological grading, it will help to adjust the aggressiveness of treatments. The molecular classification can be performed from minute amounts of DNA extracted from archived material, and is thus ideally suited for routine clinical application.
